# Enriching Future Research: The Power of Qualitative Methods in Cancer-Related Health Services Research

**DOI:** 10.1093/geroni/igaa057.2402

**Published:** 2020-12-16

**Authors:** Sean Halpin

## Abstract

The ever-changing landscape of cancer care for older adults—with novel treatments, increasing survival rates, and growing population diversity—makes effective cancer care delivery increasingly complex. Qualitative research is uniquely poised to make sense of this complexity and shape potential interventions and their implementation. While the potential power of qualitative methods in cancer-related health services research and implementation science is great, as recognized in a recent National Cancer Institute report, the range of qualitative methods can make identifying and applying the most appropriate method(s) challenging. To meet this challenge, this symposium will bring together researchers across disciplines to report on three qualitative techniques and how each was applied in cancer research with older adults. Halpin will present on the use of applied conversation analysis to study medical education delivery to patients with multiple myeloma. The method is particularly well-suited to investigate health education and communicative efficacy. Carrion will discuss in-depth qualitative interviews that were conducted to understand the cancer beliefs and attitudes of older Latinx adults. The interviews, conducted in Spanish, offer an opportunity to consider how qualitative methods are key to illuminating the experiences of underrepresented populations. Seaman will report on the multiple qualitative methods used, including questionnaires, interviews, and site observations, to document survivorship care practices among head and neck cancer programs. The triangulation of qualitative methods allowed for an unparalleled understanding of guideline implementation and program variation. Exploring a range of methods, the presentations make a powerful argument for qualitative methods in cancer-related health services research.

